# Creating year 7 bubbles to support primary to secondary school transition: a positive pandemic outcome?

**DOI:** 10.1080/03004279.2023.2186977

**Published:** 2023-05-04

**Authors:** Katya Saville, Sandra Leaton Gray, Jane Perryman, Eleanore Hargreaves

**Affiliations:** IOE, UCL's Faculty of Education and Society, London, UK

**Keywords:** Secondary school transition, COVID-19, stage-environment fit, timespace, wellbeing, adolescence

## Abstract

In this paper, we explore the benefits of new forms of in-school grouping for children moving from primary to secondary school during the COVID-19 pandemic in England. Our three-phase study with over 400 students and teachers found that protective measures to limit COVID-19 though year group ‘bubbles’ generated an environment more aligned to children’s previous primary school experience. This natural experiment smoothed the process of transition by providing a better correspondence with students’ developmental needs, especially for those on the cusp of adolescence. We recommend that physical, administrative and pedagogical school structures are reimagined for this age group to this end.

## Introduction

The negative impact of the COVID-19 pandemic on student learning and wellbeing globally across different age phases has been well reported (OECD 2021). In England, the dominant focus has been on ‘catching up’ on ‘lost learning’ (Newton et al. 2021; Rose et al. 2021) and addressing the widening achievement gap between students from disadvantaged backgrounds and their peers (Engzell, Frey, and Verhagen 2021; Cattan et al. 2021). However, schools have also had to handle a mental health emergency on top of a pre-existing mental health crisis (Patalay and Gage 2019; TES 2021), resulting in wellbeing being the pre-eminent focus for most teachers initially (Moss et al. 2021).

In England, the majority of students attend primary school from age 4 to 11, whereupon they transition to secondary school until at least age 16^1^ and, given existing research showing how primary to secondary transition is already associated with declines in achievement and engagement and increases in anxiety and depression (Jindal-Snape et al. 2020), transitioning students were of particular concern during the COVID-19 pandemic. Protective or risk factors during transition highlighted in Jindal-Snape et al’s review include individual student factors (e.g. emotional resilience, problem solving, organisational and social skills), school organisational aspects, and the degree of support from family members, teachers and both primary and secondary peers. As all three sets of factors (individual, school and social) were adversely affected by COVID-19 restrictions, it was vital to track the resulting impact on transitioning students’ achievement and wellbeing.

Initial findings from the first two phases of the research study on which this paper draws have highlighted the particularly negative impact which a lack of conclusion to the end of primary school in Year 6 (age 11) and missing standardised attainment tests (SATs) had on transitioning students (Leaton Gray et al. 2021) and how they felt they had missed out on many preparatory activities due to schools being shut from March to June 2020 in the first UK-wide ‘lockdown’, while appreciating efforts schools had made. Other studies have confirmed these findings (Ashworth et al., 2022; Bagnall et al., 2022), particularly the negative impact on social and emotional wellbeing (Nelson, Lynch, and Sharp 2021). However, in the present paper we shift our focus more forward to the experiences of these students over their first year in secondary school in Year 7 (age 11–12), during which they also experienced a second comprehensive ‘lockdown’ where schools were once again closed from January to March 2021. We therefore draw on a year’s worth of data with much larger numbers than existing studies, allowing us to see over a longer time frame how some restrictions actually appeared to create opportunities to support transition as well. In particular, we consider the impact of the implementation of government guidance to group students and teachers in ‘bubbles’ of anything from 15 to 150 students, designed to limit mixing and circulation of the virus through separate facilities or timings to use facilities.

## Background on school transition, adolescence and time

As noted above, in England, the vast majority of students experience just one major transition during their school career, at age 11, from primary to secondary school. However, due to the long history of decentralisation of school planning and provision in England, there has never been a single universal school model or standard age of transition between schools. Although England’s 1944 Education Act legislated for transfer to secondary school at age 11, no clear logic for this age was provided and there was considerable resistance (Wallace 1981), resulting in changes in the 1964 Education act which allowed local authorities to set up or formalise a three-part system, with first, middle and upper schools, meaning students transferred twice – at ages 9 and 13. By the 1970s and 80s, middle school numbers peaked in the thousands, although very few exist still today (Crook 2008). Independent and grammar schools also historically often took students at age 12, 13 or 14 and some continue to do so. Despite a recent increase in the number of all-through schools (Price 2020), the vast majority of students in England do transfer to their final compulsory school at age 11, in Year 7.

There will never be a perfect age for transition which could meet the needs of every individual. This is particularly because adolescence, as a ‘period of physical, cognitive, and social maturation between childhood and adulthood’ which is ‘loosely anchored’ (Blakemore, Burnett, and Dahl 2010) to puberty and changing hormone levels, varies widely between individuals. The average of puberty onset is age 11 for girls and 12 for boys but ranges from 8 to 15 years of age (Blakemore, Burnett, and Dahl 2010). Therefore, starting Year 7 in England tends to coincide with many being at the onset of puberty, but with others yet to enter adolescence whereas, in the past, more students would have, at age 13, already achieved the greater cognitive, emotional and social maturity required to handle challenging life events, such as school transition.

Eccles and Midgley (1989), in research in the US, showed how divergent needs in terms of cognitive maturity at different stages of adolescence can lead to a mismatch with the environment for individuals, both in and out of school. For example, female post-pubescent middle schoolers struggled with less autonomy on transition, leading to decreased motivation, attainment and wellbeing compared with fellow students yet to have gone through puberty (Eccles et al. 1993). The authors’ resulting stage-environment fit theory thus calls for adaptation of environmental contexts to suit developmental needs, although how this can be facilitated for the wide range of developmental needs within a single transitioning school cohort is an ongoing question for transitions researchers (Symonds and Galton 2014).

Wider research on transition further highlights the importance of individuals’ social contexts and Jindal-Snape (2016; 2022) builds on Bronfenbrenner’s ecosystems theory (1981) in highlighting the need to conceptualise students’ transitions as not only impacted by their ecosystem, but also as being both multidimensional and multiple. Her MMT (multiple and multidimensional transitions) theory emphasises, with the analogy of the Rubiks cube, that any change or transition experienced by one individual creates a ‘ripple effect’ (Jindal-Snape 2022) thereby impacting on the transitions of those close to them. This therefore requires researchers to also consider the transitions of those around an individual, particularly given the protective nature that positive relationships with peers, teachers and family have on transitioning students in the wider literature (Jindal-Snape et al. 2020).

Bronfenbrenner, in later work (1986, 724), supplemented his conceptualisation of micro-, meso- and macro-systems around an individual with a fourth chronosystem which, he argued, can affect all other systems indirectly in unexpected ways during both normative (e.g. school transition) and non-normative events (e.g. crises). However, the role of time in the transitions literature remains underexplored. Lingard and Thompson (2017) also highlight the need for sociologists of education to embrace a dynamic, less linear understanding of time, such as May and Thrift’s (2003) concept of ‘timespace’, whereby time and space are ‘equal partners’ in understanding the social. May and Thrift emphasise that a sense of time, or social time, is both shaped by and enacted through:

1. natural timetables and rhythms;
2. systems of social discipline; (which include spatial arrangements)
3. relationships/demarcation with technological devices; and
4. in relation to various ‘texts’ (ways we express ideas about time socially)

Therefore, we might consider how the social concept of ‘secondary school transition’ is defined in relation to not only (1) a perception of the natural rhythms of puberty and adolescence, but also (2) the formal requirements, affordances and constraints of feeding and receiving schools and (3) the formalisation of this in e.g. calendars, with (4) discussion principally constrained to Year 6 and 7 and not earlier or later in general.

Naturally, there are issues with assuming any of these aspects as being fixed and there is a danger in particular of conflating transition with adolescence, given a potential six-year span of puberty onset within any cohort. In addition, the 2020–2021 transitioning Year 6 cohort, the focus of the present study, faced unique changes to almost all social apparatus associated with schooling and beyond, as well as a complete disruption to regular demarcations of time during lockdowns. During the 18 months of our study, this cohort faced multiple, smaller transitions in and out of lockdowns as well, an example of learning time being further ‘decentralised’ (Leaton Gray 2017, 64). On the one hand, the narrative of ‘lost time’ seemed to dominate concerns about students’ attainment but, on the other hand, there was a recognition by Year 6 teachers and students, at least in our study, (Leaton Gray et al. 2021) that some time was gained by the removal of Standardised Attainment tests (SATS). This is particularly as preparation for these high stakes end-of-primary school tests in Mathematics, Reading, and Spelling, Punctuation and Grammar (SPaG), usually tends to dominate the Year 6 curriculum due to their function as public accountability measures for schools.

In summary, the COVID-19 pandemic clearly disrupted multiple aspects of transitioning students’ environmental contexts, relationships and time – and we set out to investigate the resulting impact on both wellbeing and achievement over this extended period from the end of year 6 to the end of year 7, with the aim of providing guidance for students and schools on how to support this transition more effectively.

## Research design

A mixed methods, emergent design using questionnaires and interviews was employed in order to understand both the scale and particularities of the issues affecting the transitioning Year 6–7 cohort of 2020. Both teachers and students were invited, in order to provide a range of perspectives. Initially, just one phase was planned to publish guidance for schools in September 2021, funded by Wellcome and the UCL Office of the Vice-Provost (Advancement). However, it quickly became clear that the impact of COVID on this cohort would continue into Year 7 and two more phases were planned for the first and third terms of Year 7 in 2020–2021.

### Recruitment of participants and ethical considerations

Given pandemic restrictions, recruitment moved online through departmental colleagues’ social media accounts, parental WhatsApp groups, email circulars from organisations such as the Chartered College of Teaching and, once schools reopened, Initial Teacher Education (ITE) partners. Institutional ethical approval was expedited but close scrutiny was paid to any potential time burden and triggering of negative emotions. The option of surveys or interviews was always given and working completely remotely meant both students and teachers were often at ease and interviews usually lasted longer than anticipated. All interviewers of students had enhanced DBS checks and parents were actively involved in arranging interviews. Personal data was only requested as an option to facilitate further contact.

Table 1 above highlights the initially low participation during the summer holidays and also for teachers in phase 2, where many were handling local lockdowns and temporary bubble closures. Nonetheless, rural, urban and suburban schools from all English regions were represented, with a broadly average number of students eligible for free school meals. A slightly lower than average percentage of students with additional needs were surveyed, although declaring this was optional, so possibly under-reported. No student interviewees were eligible for free school meals, so interview findings were handled with caution. Female participants were over-represented in all phases except the student summer 2020 survey. This may be expected among teacher participants (81% female) due to the makeup of the workforce, but with students the reasons are less clear. Students from several single sex girls’ schools were recruited but none from boys’ schools. It is also worth noting that student interview samples are somewhat more balanced (59% female, compared with 70% for the survey). However, it is worth bearing in mind that our study required students to access surveys online and gain parental support in order to proceed, so the voices of the most vulnerable students are likely to be under-represented in our data, something we have kept in mind throughout our analysis.

**Table 1.** Timeline of Research and participant numbers by phase.

Date	Status/phase	Teacher nos.	Student nos.
March 2020	First lockdown (Y6). Schools close.	Survey	Survey
June 2020	Phased reopening of schools.	(interview)	(interview)
*July–Aug 2020*	*First data collection round (Y6 & teachers)* Focus students finish primary (Year 6)	31 (7)	16 (5)
*Sep–Nov 2020*	*Second data collection round (Y7 & teachers)* Focus cohort starts secondary (Year 7)	22	175
Jan 2020	Full lockdown (Y7). Schools close.		
March 2021	Schools reopen.		
*May–July 2021*	*Third, final data collection round.* Focus cohort completes Year 7	56 (5)	130 (12)
	*TOTAL*	**109 (12)**	**321 (17)**

#### Data collection and analysis

Surveys were developed by considering questions from existing published studies but also to reflect the new pandemic context, with both closed and open-ended options, in line with the exploratory aims of the study, and designed using UCL Hosted REDcap software. Piloting led to slight refinements in each phase, although a consistent core of questions remained. Semi-structured interviews were video or audio recorded using UCL-hosted Microsoft Teams, with one UCL-hosted Zoom and one telephone interview due to participants’ requests. Pseudonymised transcripts were usually created within 48 h by checking auto transcripts.

Analysis after the first phase proceeded, based on Braun and Clarke’s 2006 thematic analysis, with initial themes generated deductively from existing transitions literature and survey questions in two related codebooks (one for students; one for teachers). These were amended slightly as the first few interviews were coded by two researchers separately, until agreement was reached. Open-ended survey data was likewise coded, with no new key themes emerging. Patterns of frequency in the open-ended survey responses were noted, and descriptive statistics for closed questions were included to provide indications of typicality. With subsequent phases, a few new sub-themes emerged – however, the core framework remained unchanged. Interim and final guidance was reported for students and schools and a full report from the first two phases was published in April 2021 (Leaton Gray et al. 2021). Our findings here relate more specifically to the impact of the COVID-related restrictions on students’ Year 7 experiences and also include the last phase of data in the summer of 2021.

## Findings

**Figure 1.**
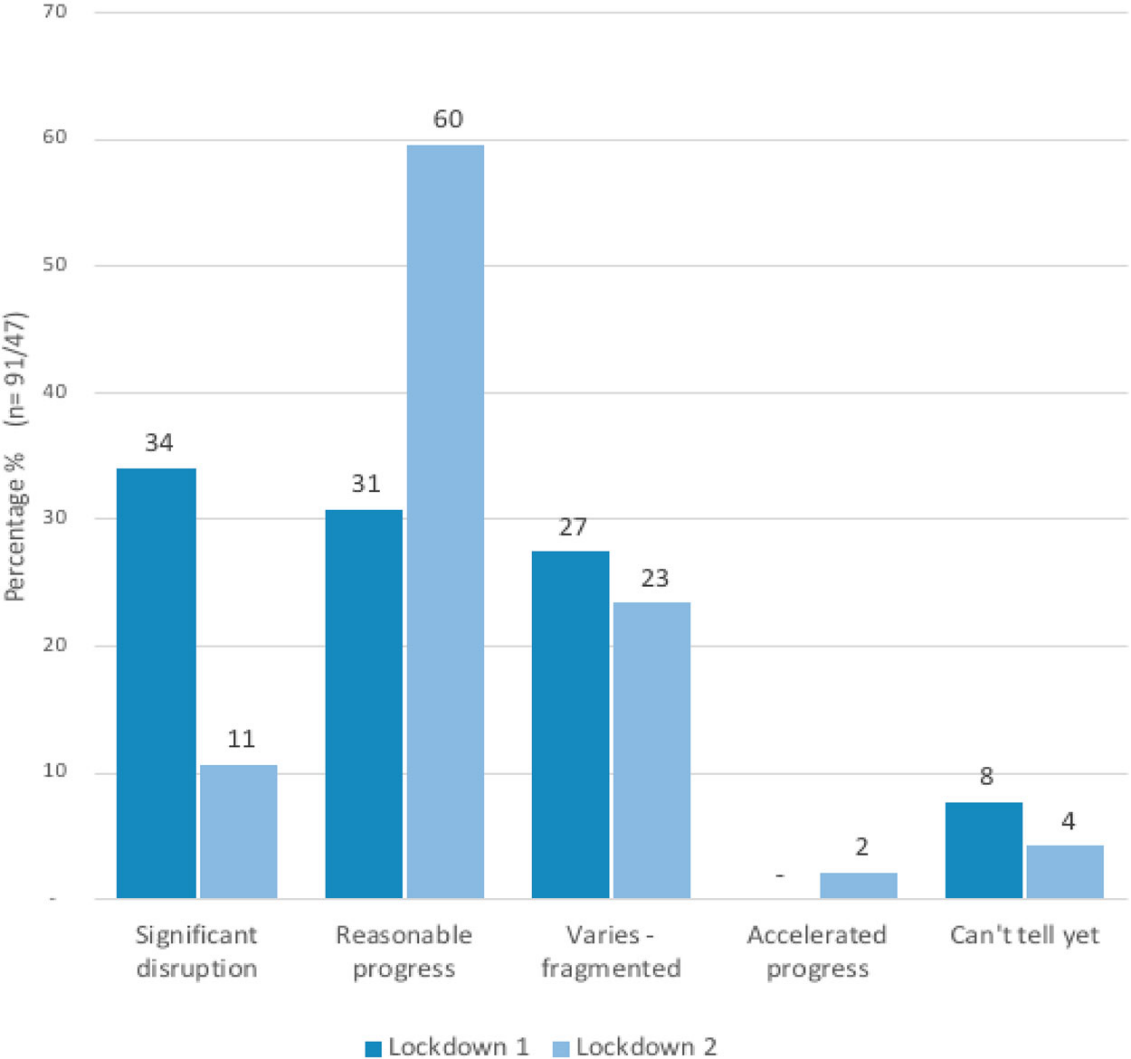
Teachers’ perceptions of scale of learning disruption in cohort during lockdowns.

The usual transition preparation and end of primary school experience ground to a halt for the 2020 graduating Year 6 cohort on 17 March 2020, when schools were closed to all but a minority of keyworkers’ children, or the most vulnerable. During the following year, most of this focus cohort spent less than four months in school and at least six months learning remotely. Therefore, it is unsurprising that our teacher participants reported significant disruption to learning for many students, particularly during the lockdown in year 6 (Figure 1).

### Learning disruption

During the lockdown in year 6, students and teachers highlighted that, not only was most learning asynchronous, much was also offline. Only 19% (54/283) of students reported having live online lessons and a number of students reported that their written work was almost never submitted for feedback.

The cancellation of SATs further disorientated, and even disappointed, many students in our study as they often felt that their hard work had gone unrecognised. However, a significant minority enjoyed exploring their own interests in greater depth and 60% (55/91) of teachers recognised that lockdown had provided students with opportunities to develop greater autonomy, while 25% recognised that students could follow more of their learning interests. Students with additional needs or caring responsibilities also appreciated the flexibility of timing provided in both lockdowns. As two students noted in the survey: ‘*Sending me textbooks/material for learning …  … allowed me to look at all the information and take my time with reading through it ‘* and ‘*in the day, I had to help with the housework so I could do my school work later in the day when I am less busy*’.

**Figure 2.**
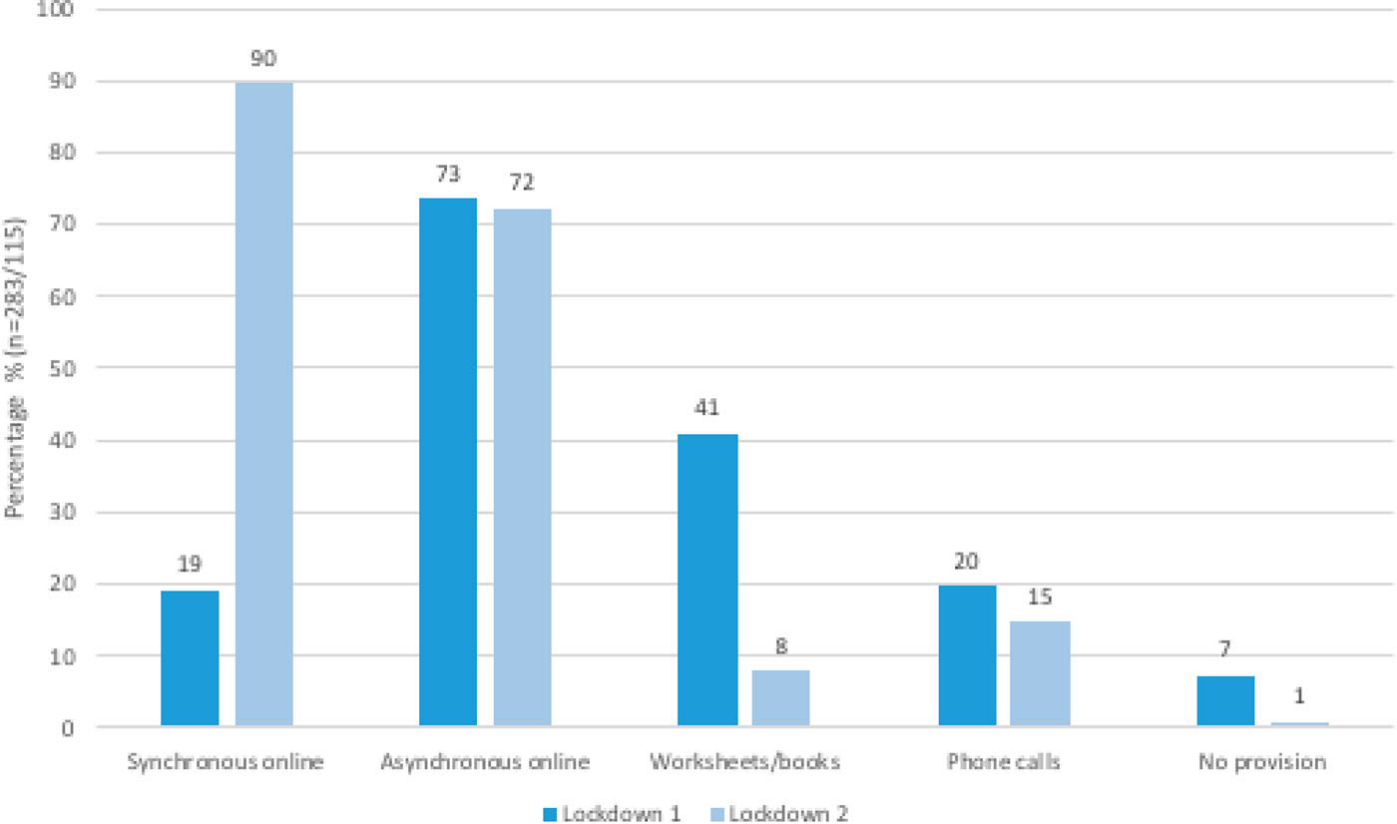
Student reports of provision by their schools during lockdowns.

The second national lockdown in 2021 (Year 7) resulted in far more synchronous online learning (Figure 2) and teachers reported far less significant learning disruption (Figure 1). In interviews, several teachers reported that they were better resourced and prepared by the first lockdown and local lockdowns and periods of isolation in Autumn 2020. However, around a quarter of teachers surveyed still reported fragmentation of the student cohort (see Figure 1), with more introverted students in particular, and some with specific difficulties, flourishing in new ways due to the online environment while others engaged very little with online provision. As Gina, a Year 7 student, noted: ‘*in lockdown, I did hardly any work …  … I just played computer games through all the lessons … … I feel like I only could start learning and being motivated again when I went back to school.’* It was also challenging to effectively monitor students’ progress, despite unprecedented efforts by school staff, such as Sophie: ‘*we were relentless … . We didn't give up on any of them and as soon as a parent said, you know, we're struggling with something, we offered them something else straight away*’.

When the Year 7 students returned to school in March 2021, teachers in our study reported that writing and test-taking ‘stamina’ had decreased, as Holly, a Year 7 teacher, noted: ‘*this week they've had the first official tests and I notice quite a few of them, even very able children who work really hard …  … they struggled sustaining the concentration’*. She, like many, also noted the multiple ‘*random gaps*’ for many, particularly in Maths. Oracy skills were also a concern.

To restrict the spread of COVID, students had been almost all organised in bubbles since the first lockdown eased, with 84% (110/131) of them in Year 7 in year group bubbles, 14% in form/class bubbles and 2% in bubbles of an intermediate size. These bubbles were usually restricted to one building or classroom for most of their lessons, according to student and teacher respondents. They also reported how the restricted use of buildings resulted in a narrower curriculum due to fewer practical opportunities or specialist equipment in many subjects. Social distancing rules also meant extracurricular opportunities were extremely limited, group work was rare and informal assessment and feedback were constrained by a need for social distancing. However, students were often stoic about the changes, as Carmen, a Year 7 student, reflects about her approach:

Year 6 kind of it all changed, so I kind of changed my expectations … … I guess I kind of knew that it wasn't gonna be completely normal, so I was like, well, we probably won't be able to do everything the exact same as it worked out, so I kinda just went in open minded like, well, it's gonna be different.

More importantly, to our surprise, the students and their teachers also revealed some unexpected benefits of being in their bubbles.

### Bubbles – an extension of primary?

In spending most of Year 7 with the same class of around 25–30 students in the same room, for many students, starting secondary school ended up being more similar to Year 6 than expected. There appeared to be several ways in which the restrictions actually sheltered students from aspects which they said they had been concerned about. By being limited to one or two buildings, many were happy not to get as lost as they expected. As one student noted in the survey during their first weeks in Year 7:

You can’t do a lot of things like go in the science lab or art room but for me because all your lessons are in one building it feels nicer.

Even by the end of Year 7, 32% (42/131) were still pleased to be mainly based in one classroom. However, the majority were keen to ‘explore’ other classrooms and buildings by this point. As Gina, a Year 7 student, reflected when asked whether bubbles should continue,

If the bubbles are kept in place, then the Year Sevens, some of them who were sort of worried about the older years, could feel more comfortable. However, the ones that want to like be more, you know, ambitious and more explore-y can't really do that as much.

But overall, many did appreciate the smoother transition bubbles afforded, at least for a short time. As a student reflected in the survey at the end of Year 7:

Coronavirus makes it seem like primary because we would stay in the same classroom for every lesson so it has eased the transition and it will be easier hopefully soon to move around and discover the school. On the first day, I wouldn't want to encounter a year 11 but now, it would be much easier and I have adapted to secondary life.

Outdoor space was also limited, with most students reporting reduced time, space and play equipment than expected at break and lunchtime, particularly compared with their pre-lockdown, primary experience. Ball sports were sometimes permitted but usually impossible. As Jack, a Year 7 student, noted:

We can't do anything really except just talk ‘cause there's like no grass, so we can't play football …  … there's not a lot of room, like if you're trying to run around, you’re just probably just gonna run into something.

Although some clearly felt frustrated by this, many students, particularly girls (but by no means all), seemed content to chat in small groups rather than engage in physical activity. This might be expected as many girls enter adolescence sooner than boys and are perhaps therefore more comfortable with less active play. Overall, this does suggest that bubbling did not facilitate an extension of primary-style play in the main, perhaps particularly due to a lack of space and equipment. However, with play restricted to Year 7 peers, there is some evidence that bubbling facilitated more imaginative play for some. As one teacher reported: ‘*I still had Year Sevens playing almost make believe … dungeons and dragons … You don't normally get that in secondary*’. Many students also appreciated the staggered lunch arrangements to reduce year group mixing, which many teachers reported would stay post-pandemic.

Setting or streaming by ‘ability’ or attainment was suspended or limited for many Year 7 students. Although some difficulties and disappointments with the resulting mixed attainment learning spaces were reported by a few participants, there were indications that many students benefited, with one student survey respondent noting ‘*it is better to stay in one class with all of my classmates rather than be separated by ability*’. Tammy, a high achieving Year 7 student, also appreciated a whole class, mixed attainment approach to ‘catching up’ when noting on returning after lockdown: ‘*I had been doing the work, but I still hadn't really got the opportunity to ask that many questions, but then we went over it back again at the end. I thought it was just like a relief*’. Reviews of research on ability setting have shown that, although there is evidence of a small positive benefit in outcomes for higher attainers, there is zero or negative impact on outcomes for lower attainers (EEF 2021). Therefore, a mixed attainment classroom post-lockdown would appear to provide potentially greater benefit for lower attainers than removal. Only a few of our participants had experienced separate ‘catch up’ classes and were not very forthcoming in their opinions about these but Cally, a Year 7 student, did note some negative impact when stating of teachers: ‘*they think, oh, Maths is more important, so it’s like, they couldn't take you out of maths, English or science, but they would take you out of something like music.’* Rather than removing struggling students for separate catchup classes, the incidental mixed attainment grouping approach experienced by many in our study appeared to provide benefits for a broader group of learners, perhaps especially given the ‘*random gaps’* which teachers had reported that many students had experienced as a result of lockdowns.

As we have discussed earlier in the paper, in England, school transition at the age of 11 has come to be viewed by many as a rite of passage into adolescence as well, albeit using a somewhat arbitrary timepoint (Boyle 1972). This is reflected, for instance, by the majority of students in our study noting how their play had abruptly become less physical and that they (or their friends) spent time simply chatting but, as one student respondent noted, ‘*I’m ok with that*’. The slightly problematic conflation of starting secondary school with adolescence, when many students are actually biologically (and therefore, to some extent, emotionally and socially) yet to reach adolescence, we would argue, makes the primary to secondary transition at age 11 inherently problematic, due to lower likelihood of ‘fit’ between school and student for many this young. However, what our data indicates is that, by bubbling these transitioning students in 2020 and 2021, a natural experiment occurred which inadvertently stretched the primary phase in many ways. This meant that the systems of social discipline which May and Thrift (2003) highlight (particularly spatially both within and outside of school in lockdown) and technological demarcation of time (timetables and calendars reformulated during lockdowns) associated with the usual timespace of transition were completely disrupted or even suspended in terms of integrating into the full life of the secondary school. In this sense, bubbles also challenged the common narrative of transition ending in Year 7, with several teachers and students referring to Year 8 as involving a sense of transition for this cohort. For instance, isolation from other year groups, was, by the end of the year, something which both teachers and students were keen to reverse. However, the relative isolation created by bubbles also appeared to have positive impacts on in-year relationships and wellbeing.

### Benefits of bubbling for mental health and relationships

74% of teachers surveyed (68/92) had increased concerns about their students’ mental health because of the impact of COVID and reported particular issues with social isolation amongst Year Seven students, as Linsey, a head of Year 7, captures here:

I think that's quite unique to Year Seven, and perhaps that is because they've never really had the time to settle in at school. So I think there's a lot of anxiety and a lot of kind of worries about friendships and bullying.

The cumulative effect of two lockdowns in two different schools appeared to particularly threaten transitioning students’ usually protective peer relationships. As Maddie, a Year 7 teacher, noted:

They're eleven. They’re only really just into school when suddenly it wasn't. We had one term where things were a bit weird with bubbles and they had to wear masks … and then suddenly they were thrown back home. So I know that they struggled friendship-wise because … they've only had three months to develop friendships. They’re not strong enough to keep going.

More than one student noted how they had neither developed good enough friendships – nor exchanged contact details – with their new Year 7 classmates prior to the second lockdown, in addition to not being able to sustain Year 6 relationships, due to not returning to primary school after the first lockdown. As Jonny, a Year 7 student, reflected:

One of the big issues, I wasn't really able to re encounter with many of the people in my primary school after the lock down …  … I feel kind of like upset about it because like there were loads of people that I used to know, and I just don't really know them anymore.

Therefore, in line with existing research in primary settings (Moss et al. 2021), it is unsurprising that most schools prioritised Year 7s’ mental health over learning both during and after each lockdown. As head-of-year Linsey remarked, ‘*making sure that students are well and are happy and are settled is by far the biggest priority in terms of actually addressing those academic concerns later on’*. A number of the teachers we spoke with clearly did everything possible to encourage friendships, providing virtual buddies and/or using live online lessons to create a social space which translated, in teacher Becky’s case, to increased confidence and belonging for a student with SEN:

I have a child who is very, very severely dyslexic and has real issues with his motor skills …  … School has always been really challenging for him, and when I asked who wanted to write the next quiz for the next social, this tentative little hand went up and he went away and he came back the next week. He was so proud he'd created this quiz all about sharks and all the children who completed this quiz were like ‘I have no idea how to answer this question about some shark’ and you could just see him smile because he knew something that they didn't and he'd created this quiz that they were finding really challenging.

The impact on this student’s learning was still evident months later, as Becky continued:

He was telling me the other day that Lyra from the Northern Lights is really naughty and rebellious. And I was like ‘yeah, she is Matthew, that's a great catch,’ and he was like ‘yeah she was smoking,’ and it’s really nice seeing him engaged.

It is worth noting that Becky and Holly were teachers who spent several hours daily with the same class, in schools with Year 7-specific models – so increased time with their forms was already a planned feature of their schools. Of course, as we noted above, bubbling restricted and limited some friendships, with 35% (46/131) of students surveyed saying they missed seeing friends outside their bubble. Therefore, ultimately, 62% of those surveyed didn’t want COVID bubbles to remain. However, 26% were clear that they did want some aspects to continue, with 30% overall saying they enjoyed the extra time bubbles had provided with their classmates. Carmen, a Year 7 student, in interview, highlights this tension in noting both that: ‘*my friends in other classes, we kind of grew apart … *’ but also later: ‘* … I think our form’s probably closer than it would have been in other years because we kind of almost, not been fully isolated from everyone else, but we kind of met each other and like we’ve grown quite close*.’

Bubbling also meant more time with one or two dedicated adults for many, like in primary school, in order to limit circulation of staff. We suggest that this approach may be partly responsible for the fact that many students expressed surprise at how kind and approachable their teachers, particularly form tutors, were, compared with their expectations of multiple, strict secondary school teachers.

Multiple students told us they felt less worried about bullying, as they were unlikely to encounter older students, particularly in crowded corridors, which removed what is a common concern of many transitioning students (West et al., 2010). However, this also had negative consequences too, beyond merely the desire of some to branch out more. As one student in our survey reflected, it simply shifted the problem somewhat: ‘*I couldn't interact with other year groups, making it more awkward whenever I saw them’.* Several teachers also noted that the Year 7s had fewer role models and therefore appeared more confident, yet less ‘mature’, than previous cohorts. The flipside of this was, however, that quite a lot of students noted closer bonds with their peers, as noted above.

In recent years, mental ill health among those going through adolescence and transition is an increasing concern (Blakemore 2019; Evans, Borriello, and Field 2018). Drawing on our findings, we suggest that perhaps these concerns are, in part, the reflection of a fundamental lack of fit between many secondary school environments and the developmental needs of many beginning Year 7 students, as the transition at age 11 occurs for many before the biological age of onset of adolescence and its associated social and emotional maturational changes. Our findings here suggest that, where more aspects of the lower secondary school environment (particularly relationships) are similar to primary schooling, this provides a timespace where more students are able to develop the necessary social, psychological and emotional skills needed to successfully navigate their transition to secondary school.

### The potential impact on academic attainment

Some teachers in our study reported that their students quickly ‘caught up’ on learning once back at school post lockdown, despite restrictions. As noted above, Holly worked in a school which already had a staggered approach to transition, with specialist teachers who are primary trained and teach several subjects. Therefore, each Year 7, mixed attainment form has the same teacher for one to two hours a day. Holly noted the importance of this continuity when she reported of remote learning in 2021 that:

You felt like you were in it together, you were a team. I think that the relationship with this year group is very, very strong and I think that's why, when they came back, they had this safety net and they just attacked their learning. They really did. I feel they’re fearless and that's across the board. Even my very vulnerable children.

We might contrast this with Dominic, a Year 7 student, who attends a school without a Year 7 specific approach and remarked that his teachers ‘*even a year through, some of the subjects that you have very rarely, they’re still trying to catch up on names*’. However, we above acknowledged that the majority of students were pleasantly surprised by their Year 7 teachers and the efforts made to connect with them, especially during lockdown. Nonetheless, having an institutional structure such as in Holly’s school, appeared to foster particularly strong relationships even through remote learning periods, and it was this unity which allowed students to focus on learning and flourish, despite the same negative influencing factors presented by the pandemic. We suggest that these experiences, also reflected in teacher Becky’s school, and student observations on how they appreciated the mixed attainment classroom, add further weight to questions around the suitability of withdrawing students for catchup provision. It instead suggests that prioritising a primary-style, mixed attainment approach, similar to that created naturally by COVID bubbles, actually facilitates the very protective positive peer- and teacher- relationships which are associated with smoother wellbeing and better academic outcomes for transitioning students.

## Recommendations

What can we learn from the natural experiment of a transition supported by bubbles, now that the pandemic is easing, and this year’s new secondary cohort in England has had a year free of lockdowns? We are certainly not recommending bringing back a later school transition. This is because the model of lower, middle and upper schools (with transition at ages 9 and 13) has struggled to retain a foothold for a variety of reasons, including accountability issues created by a mismatch between the points of transition and government standardised ages of testing (at age 7, 11, 14 and 16) (Crook 2008, 123). We are also not advocating necessarily for expansion of the all-through school sector with no institutional transition at all, as these are still quite innovative in the English context and therefore require greater long-term evaluation. Finally, we cannot recommend some kind of permanent COVID-style schooling. Restricting students to one classroom and/or one form of 30 students would not be supported by our findings and, indeed, very few participants experienced this extreme implementation in secondary school. All students we surveyed and interviewed noted that bubbles were restrictive in some regards and this was very problematic where there was a lack of stage-environment fit in terms of autonomy (Eccles et al. 1993), for those who wanted to ‘explore’ more. Of course, these students will have now been able to meet older students as restrictions have lifted, as well as through shared opportunities such as extracurricular clubs, so many of their fears will have been relieved.

We do, however, recommend that the benefits of bubbles for the majority of students may be significant, particularly for those who struggle with the multiple social, practical, emotional and cognitive demands of starting a secondary school environment designed for older adolescents. Their needs are likely to be better met by ensuring that the timespace in which transition occurs is made more developmentally appropriate and flexible in small ways. Recommendations already adopted by some schools in our study, or who have been in correspondence with us subsequently, include discrete Year 7 facilities, such as one floor of a building with their own toilets, pastoral support offices and a specific library or sitting area. Specified times, equipment and/or spaces for play with Year 7 peers might also facilitate developmentally appropriate play. Year 7 specific teachers, often primary trained, who teach mixed attainment classes in one classroom for up to several hours a day, also allow peer and teacher relationships to be closer to those of primary school with the concomitant advantages of mixed attainment teaching better supporting lower attaining students’ attainment outcomes.

The discourse for a number of years now in primary schools, particularly regarding Year 6 academic achievement, has been on ensuring pupils are ‘secondary school ready’ (BBC 2022) but what our data demonstrates is how important it is that all schools are Year 6 and 7 pupil ready, particularly in their nurturing of peer and teacher relationships and catering for pre-adolescent students’ developmental needs. There is a clear concern regarding equity of transition processes here as some students (particularly female) are entering puberty right at the point of starting school, and research shows the detrimental effect that additional changes can have during this vulnerable time (Ge, Conger, and Elder 2001). However, other research (Jordan, McRorie, and Ewing 2010) suggests that those who are more mature emotionally find transition easier, so it may be that those yet to enter this phase are more likely to struggle, particularly given the mismatch of an environment which is aimed at older adolescents in general. In particular, male students yet to enter puberty may well benefit more from being sheltered from older, larger, students and having more age-appropriate play facilities, although several female students in our study also missed more physical play. In sum, we recommend challenging the current timespace of secondary school transition with some small, carefully planned aspects of primary schooling for an interim period, while introducing opportunities to encounter secondary schooling at students’ pace.

Unlike during the pandemic, Year 7 students should be free to circulate in other areas of the school, or choose to use their own dedicated facilities. Furthermore, a range of extracurricular activities should be made available for all, benefiting particularly those who are ready to engage with older students. We also recommend a staggered induction for the entire Year 7 cohort to whole school activities, as many schools already provide, through cross-year group house activities and buddy systems, where Year 7 students are given a buddy in an older year group. This would, we argue, go some way towards a more equitable transition for greater numbers of students.

## Note

1 Some may attend separate infant and junior schools from age 4–7 and 7–11 respectively instead of a primary school and a very small, but growing, minority may attend all-through schools from 4–16 or 18. However, in a few regions of England, a three-tier system persists, whereby students transition from first to middle school at age 9 and from middle to upper school at age 13. However, this is declining (see below)
